# Projected Uptake of New Antiretroviral (ARV) Medicines in Adults in Low- and Middle-Income Countries: A Forecast Analysis 2015-2025

**DOI:** 10.1371/journal.pone.0164619

**Published:** 2016-10-13

**Authors:** Aastha Gupta, Sandeep Juneja, Marco Vitoria, Vincent Habiyambere, Boniface Dongmo Nguimfack, Meg Doherty, Daniel Low-Beer

**Affiliations:** 1 Medicines Patent Pool, Geneva, Switzerland; 2 Department of HIV/AIDS, World Health Organization, Geneva, Switzerland; University of Pittsburgh, UNITED STATES

## Abstract

With anti-retroviral treatment (ART) scale-up set to continue over the next few years it is of key importance that manufacturers and planners in low- and middle-income countries (LMICs) hardest hit by the HIV/AIDS pandemic are able to anticipate and respond to future changes to treatment regimens, generics pipeline and demand, in order to secure continued access to all ARV medicines required. We did a forecast analysis, using secondary WHO and UNAIDS data sources, to estimate the number of people living with HIV (PLHIV) and the market share and demand for a range of new and existing ARV drugs in LMICs up to 2025. UNAIDS estimates 24.7 million person-years of ART in 2020 and 28.5 million person-years of ART in 2025 (24.3 million on first-line treatment, 3.5 million on second-line treatment, and 0.6 million on third-line treatment). Our analysis showed that TAF and DTG will be major players in the ART regimen by 2025, with 8 million and 15 million patients using these ARVs respectively. However, as safety and efficacy of dolutegravir (DTG) and tenofovir alafenamide (TAF) during pregnancy and among TB/HIV co-infected patients using rifampicin is still under debate, and ART scale-up is predicted to increase considerably, there also remains a clear need for continuous supplies of existing ARVs including TDF and EFV, which 16 million and 10 million patients—respectively—are predicted to be using in 2025. It will be important to ensure that the existing capacities of generics manufacturers, which are geared towards ARVs of higher doses (such as TDF 300mg and EFV 600mg), will not be adversely impacted due to the introduction of lower dose ARVs such as TAF 25mg and DTG 50mg. With increased access to viral load testing, more patients would be using protease inhibitors containing regimens in second-line, with 1 million patients on LPV/r and 2.3 million on ATV/r by 2025. However, it will remain important to continue monitoring the evolution of ARV market in LMICs to guarantee the availability of these medicines.

## Introduction

The number of people on antiretroviral therapy (ART) in low- and middle-income countries (LMICs) continues to grow, with the number of people receiving ART reaching 17 million by end 2015 [1]. Currently 95% of people taking ART are residing in LMICs [2]. 36.9 million people globally need ART but almost half of them are not yet accessing it, leaving treatment coverage still well below the 90% target proposed by UNAIDS in 2014 [3]. Despite the gap in ART coverage and constraints in international donor funding however, UNAIDS has stated that the resources to support the fight against AIDS will continue to increase and ART scale-up will also likely continue, at least over the next few years [3,4]. Demand for ART globally is not levelling off, and it will remain crucially important that manufacturers, global policy planners and procurement agents anticipate future changes to treatment regimens, demand, and the generics pipeline, in order to secure continued access to all antiretroviral (ARV) medicines needed.

Therapeutic innovation will undoubtedly lead to major shifts in the composition of the treatment regimens used in the near future, because safer, more effective, cheaper and easier to use medicines and formulations are being developed. For instance, ViiV Healthcare obtained market approval for DTG in the USA in August 2013 [5] and in Europe in January 2014 [6]. Gilead Sciences obtained approval from the US Food and Drug Administration (USFDA) for a novel and less toxic prodrug of tenofovir called TAF in combination with emtricitabine (FTC) in April 2016 [7]. Generic manufacturers have already been granted patent licences for these novel drugs and are already exploring and developing fixed-dose combinations (FDCs) involving TAF and DTG. Furthermore, there are multiple novel ARV drugs in Phase III of clinical development including new class of drugs that, if successfully developed, could benefit both the patients and funders.

While most people in high-income countries will be able to access these new ARV drugs and formulations as soon as they have been approved by their regulatory authorities, patients in LMICs usually will have to rely on the availability of affordable generic drug formulations because they will not be able to pay the usual high originator price. In addition, patients in LMICs often have to wait for WHO and their national guidelines to recommend the use of new drugs and regimens. WHO approves the use of newer drugs typically with some delay because—unlike drug regulatory authorities in high income countries—WHO considers the affordability and availability of generic FDCs as an important aspect in its treatment guidelines. However, because voluntary licenses for DTG and TAF have already been obtained by several generic pharmaceutical manufacturers through the Medicines Patent Pool [8], multiple generic versions of DTG might become available as soon as 2017, and generic formulations containing TAF by 2019. Once affordable generic versions of these drugs are available, the mix of ARVs used in LMICs will change considerably.

In order to anticipate the changes and to secure continuing access to all ARVs needed (first, second, and third-line regimens), we did a forecast analysis to estimate the likely number of PLHIV taking a range of new and existing ARV drugs in LMICs up to 2025, and their market share. We analysed the following ARVs: atazanavir/ritonavir (ATV/r), darunavir/ritonavir (DRV/r), dolutegravir (DTG), efavirenz (EFV), lopinavir/ritonavir (LPV/r), nevirapine (NVP), raltegravir (RAL), tenofovir alafenamide (TAF), tenofovir disoproxil (TDF) and zidovudine (AZT). These drugs are typically used in combinations of three including nucleosides/nucleotides such as AZT, TDF, and TAF, in addition to 3TC and FTC—which we define as “backbone” drugs—and non-nucleoside analogues (EFV, NVP), protease inhibitors (ATV/r, DRV/r, LPV/r) and integrase inhibitors (DTG, RAL)–which we define as “companion” drugs.

## Materials and Methods

To estimate the number of people taking each ARV medicine up to 2025 in LMICs, we projected both the total number of people taking ARVs in each year and the market share of each ARV drug in the mix of regimens used (first, second, and third-line regimens). LMICs were defined using the World Bank Atlas definition [9]. The data on total number of people on treatment from 2016–2025 was obtained from John Stover (Avenir Health) and Peter Ghys (UNAIDS) and comprised data used for the UNAIDS Fast track report [3,4] and their published study [10]. The data on past market share of each ARV was obtained from presentations on the WHO Survey on ARV Use, 2010–2015 [11–16] to obtain a trend of market use for all ARVs. These data were compiled in an Excel file (S1 FC) and then a modeling approach was used to predict market shares for each drug in each line of treatment.

### Calculation of total number of people on ART

We drew on secondary data sources to calculate the projected total number of adults in LMICs taking ARVs (first, second, and third-line treatment regiments) from 2015 to 2025. We used the number of patients currently on treatment [17] and the targets of the Fast Track Scenario proposed by UNAIDS in 2015 [4]. These data assume that at the end of 2025, there will be 28.5 million adults in LMICs on ART, 14.5% of whom will be on second- or third-line regimens by 2025 (from 4.2% at baseline in 2015). We used the Fast Track Scenario [4] estimate for adults on first-line treatment without modification. However, because the Fast Track Scenario does not state the number of adults likely to receive third-line therapy, we split its projected number of adults on second-line treatment into adults receiving second-line, and adults receiving third-line treatment, assuming that the same proportion of adults switching from first to second-line treatment would switch from second-line to third-line treatment. This assumption is in line with rate of resistance to protease inhibitors shown in some second-line studies [18].

### Modeling the uptake of new ARV drugs (DTG and TAF)

To derive the use or uptake of new ARV medicines in LMICs, some key assumptions and variables were used, as follows:

1. Likely dates at which each ARV would be available to people in LMICs through multiple generics on the market: Data were acquired through the Medicines Patent Pool (MPP), compiled from close interaction with generic manufacturers who reported that: more than one generic version of DTG tablet, single drug formulation, will be introduced following approval by stringent regulatory authorities or WHO prequalification by the fourth quarter of 2017; DTG containing FDCs are expected to be introduced by third quarter of 2018; and TAF containing FDC by first quarter of 2019.
2. Potential price: Launch price of DTG and TAF are assumed to be close to the prices of EFV and TDF, respectively, at the time of introduction. Post-launch the new ARVs will follow historical price erosion trends
3. Clinical study: Favorable study results on safety and efficacy of DTG and TAF in specific populations (TB/HIV co-infected patients, HIV-infected pregnant women) are expected to be available and the potential issue of drug interaction of TAF with rifampicin and efficacy during the last trimester of pregnancy will have been resolved.
4. WHO Guidelines on ARV Use: recommend new ARV products, first as an alternative and then after 2–3 years as part of the preferred ART regimen
5. National Guidelines: New products included on national guidelines within 1 year following publication of WHO Guidelines on ARV Use

To project future use for any new ARV, we generated a set of uptake curves and then used these to project future demand to 2025 (Table 1).

**Table 1:** Cumulative proportion of market share for new drugs under each uptake curve.

Uptake Curve	Year after introduction										
	Y0	Y1	Y2	Y3	Y4	Y5	Y6	Y7	Y8	Y9	Y10
Conservative (25%)	0%	1%	2%	3%	4%	5%	6%	7%	8%	10%	11%
Conservative (50%)	0%	2%	4%	6%	9%	11%	13%	15%	17%	19%	21%
Conservative (75%)	0%	3%	6%	9%	13%	16%	19%	22%	25%	29%	32%
Baseline (100%)	0%	4%	8%	12%	17%	21%	25%	29%	33%	38%	42%
Aggressive (125%)	0%	5%	10%	15%	21%	26%	31%	36%	41%	48%	53%
Aggressive (150%)	0%	6%	12%	18%	26%	32%	38%	44%	50%	57%	63%

Y = Year.

**Fig 1:**
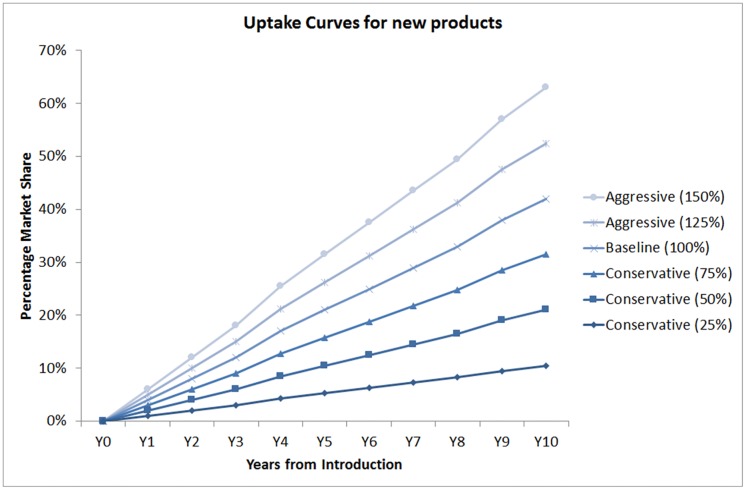
Uptake curves: cumulative proportion of market share for new drugs under each uptake curve.

We did this by looking at historical data of market share trend of TDF from the Global Price Reporting Mechanism [19] during the period 2003–2012 –which is from the date of introduction of TDF in LMICs up to 10 years. These data provide information on actual purchase of each product through major procurement agencies in LMICs. The data for 2003–2012 showed a very slow uptake of TDF in the initial 6 years and then an exponential growth as the product became generically available and became the preferred regimen in WHO Guidelines on ARV Use—at 0% in 2003 and 42% in 2012. However, taking into consideration the current market scenario where generic versions of new products are likely to be available early on in LMICs due to voluntary licences obtained by the MPP, this real historic data was then converted into a near-linear trend line (which we call “baseline” in Table 1). This baseline trend of uptake was then extrapolated to construct more aggressive or conservative uptake curves at 150% and 125%, and more conservative uptake curves at 75%, 50%, and 25% of the corresponding TDF market share in a particular year (Table 1; Fig 1).

We made the assumption that each new ARV drug would follow one of these uptake curves at any point in time, and that the uptake curve followed by that drug could change as a result—for example—of the competitive situation, pricing, availability of fixed-dose combinations (FDCs), and inclusion in WHO Guidelines on ARV Use.

### Extrapolating data on use of existing ARVs

We extrapolated data presented on current global use of ARVs from the annual WHO Survey on ARV Use, which covered years 2009, 2010, 2011, 2012, 2013 and 2014 and included 94%, 86%, 53%, 62%, 74% and 91% of people on ART in LMICs, respectively [11–16]. We extracted data on the market share of different ARVs in adult ART in these years from the presentations and made a statistical extrapolation—using generalised additive methods—of the market share of different molecules up to 2017 for ARV drugs used in first-line, second-line, and third-line treatment separately. We were therefore able to modulate the extent to which older molecules will be replaced with newer ones using one of the uptake curves shown in Table 1. Within all treatment regimens we did this analysis separately for backbone and companion drugs.

### Detailed assumptions of projected ARV use in first-, second- and third-line treatment

We made the following assumptions for each uptake curve used for the new products—DTG and TAF—and also projected DRV/r in second line treatment, although it is currently only being used in third-line treatment. Guidelines on the use of these ARVs is defined in the WHO Guidelines on ARV Use 2016 [20], where DTG is a part of the alternative treatment regimen in first-line and DRV/r is a second-line treatment. Although TAF is not yet in the WHO guidelines, it has the potential to improve treatment and make it less expensive and so we included it in our analysis. Subject to availability of clinical data and regulatory approvals, it may become a candidate for inclusion in guidelines in the future.

### Modeling the projected use of ARV in first-line treatment

#### Backbone drugs

With respect to backbone drugs, the WHO Guidelines on ARV Use 2006 [21] recommended TDF as the preferred first-line nucleoside/nucleotide option and the first tenofovir/lamivudine/efavirenz generic formulation was approved in 2009. Following the availability of generic tenofovir containing FDCs, TDF has gained market share from AZT, the previous preferred first-line drug. Using these data, our projections show a continuing decline in use of AZT in first-line treatment.

The data we used to make a case for replacement of TDF with TAF in our model is: (i) TAF is a prodrug (defined as a medication that, after administration, is metabolized into a pharmacologically active drug) of tenofovir, as is TDF. TAF achieves higher intracellular concentration of tenofovir diphosphate (the active drug responsible for antiviral activity) and lower circulating plasma concentration of tenofovir compared with TDF, making it non-inferior to TDF at a fraction of the dose, with less bone and renal effects; (ii) TAF is similar in effectiveness to TDF [22]; (iii) TAF has superior toxicity profile compared with AZT and TDF [23–26]; and (iv) TAF requires only 25mg of active ingredient/day (compared to 300mg/day for TDF and 600mg/day for AZT)–this will make the tablets smaller and therefore easier to take.

Although TAF is projected to be priced at par with TDF at the time of launch, TAF-containing tablets may eventually be less expensive than those containing TDF or AZT—because the cost of the active ingredients is the main cost driver of the price of generic ARVs [27].

In our model we assumed that TAF will be introduced in year 2019 and following that will begin replacing TDF. In the initial 3 years (Year 0 to Year 2), we project that TAF will follow the 50% uptake curve (Table 1) due to the lack of clinical data on use of TAF with a WHO recommended companion drug such as EFV or DTG. Studies on use of TAF with DTG are being planned but they will take time and if successful, further time would be needed by generic manufacturers to develop FDCs with TAF. We made the assumption that if this goes well, such FDCs will be available by 2019 and use of TAF will begin picking up significantly by year 2024 once generic FDCs have approved by stringent regulatory authorities and national drug regulatory authorities. In addition, projections were made on the basis that there is currently a lack of clinical data for pregnant women, and uncertainty about safety of TAF in TB patients on account of its interaction with rifampicin, which is one of the main reasons we have assumed a late introduction of TAF. While pharmacokinetic studies to clarify this issue are ongoing, we have been guarded in our approach until their pendency. Lastly, we based assumption on the fact that patients who are stable and doing well on TDF will likely not be switched to TAF.

However, after the initial low uptake, for our forecast model we have assumed that TAF will follow the 125% uptake curve for Years 3 & 4, and then move up further to the 150% curve. These higher projections are based on the projected lower price of TAF versus TDF as well as the assumption that positive study results will be available on use of TAF with TB/HIV co-infected patients and HIV-infected pregnant women.

#### Companion drugs

With respect to companion drugs, NVP has already been replaced by EFV as the current market leader in non-nucleosides in first-line treatment because it is the preferred companion drug now according to the WHO Guidelines on ARV Use 2016 [20]. We continue the declining trend of NVP in our projections.

EFV, in turn, is expected to be replaced increasingly by DTG from 2019 onwards. The case for replacement of EFV with DTG is enumerated as follows: (i) DTG is more effective than EFV and NVP [28,29]; (ii) DTG is less toxic that both EFV and NVP [30,31]; (iii) DTG requires only 50mg of active ingredient/day (compared to 600mg/day for EFV and 400mg/day for NVP)–this will make DTG-containing tablets smaller and therefore easier to take. It is important to note that in the case of TB co-infected patients using rifampin, the effective DTG dose is under evaluation and expected to be doubled and used in a twice-daily schedule; and (iv) DTG-containing tablets will eventually be less expensive than those containing EFV or NVP, because the cost of the active ingredients is the main cost driver of the price of generic ARVs [27]

We projected that starting from 2016 as Year 0, DTG will replace EFV, during the initial 3 years at 50% replacement scenario, in next 3 years at 125% replacement and thereafter at 150% replacement. We made the assumption that the initial uptake of DTG will be 50% based on the fact that in its WHO Guidelines on ARV Use 2016 [20], WHO recommends DTG as an alternate to EFV and not as the preferred option (even though DTG price will be comparable to EFV). In addition, we based our assumptions on that fact that there are limited data available for DTG use in TB/HIV co-infected patients and pregnant women, DTG will not be available as an FDC with other WHO recommended ARVs, and lastly that there is a lack of registration of DTG in LMICs.

We then made the assumption that by Year 3 the uptake rate could be 125% due to the availability of clinical data on TB co-infected patients and pregnant women, the availability of generic FDCs such as TDF/FTC/DTG and TDF/3TC/DTG, a reduction of price of single agent DTG 50mg tablets, and an assumed shift from alternate to preferred ARV in the WHO Guidelines on ARV Use 2016 [20].

Thereafter, from Year 6, the uptake rate could be 150% since there would be a price drop of generic FDCs, more national approvals and widespread country level use.

### Projected use of ARV in second-line treatment

#### Backbone drugs

With the increased use of TDF in first-line regimens, the use of AZT is projected to increase in second-line regimens with patients coming off TDF in first-line and being offered AZT in second line. Use of TAF would be limited in second-line regiments and follow a 50% uptake curve for use in combination with protease inhibitors till 2025, mainly due to the low use of tenofovir (both TDF and TAF) in second line. DTG can also be used by patients in second line who have not used this drug in first line. However, because this approach for DTG is not yet recommended in the WHO Guidelines on ARV Use 2016 [20], at the moment we project that use of DTG in second line would be only at 75% uptake curve from 2016 till 2025, likely replacing TDF and AZT. Because there would be increased use of DTG in first- and third-line treatment, its use in second-line would remain limited.

#### Companion drugs

Currently the two preferred drugs for second-line regiments according to the WHO Guidelines on ARV Use 2016 [20] are ATV/r and LPV/r. According to the WHO Survey on ARV Use 2014 [15] and 2015 [16], ATV/r is progressively taking away market share from LPV/r. We have assumed in our model that this trend will continue till 2025. We project the introduction of DRV/r as a heat stable co-formulation in year 2019 with a 25% uptake curve and that it will take the market share from LPV/r. In spite of better virological response and tolerability than LPV/r [32–34] and better tolerability than ATV/r [35,36], DRV/r’s predicted large pill size, higher dosage (800mg once daily vs 300mg once daily for ATV/r) and elevated price (due to higher dose and more complex chemical synthesis compared with LPV/r and ATV/r) will undoubtedly remain a setback for the expanded use of this drug.

### Projected use of ARV in third-line treatment

We made the assumption that starting from 2016, driven by once daily dose, lower cost, and wider generic availability (due to voluntary licences through MPP effectively covering up to 131 LMICs), DTG will replace RAL following the 125% uptake curve for initial 5 years. It would then drop to the 100% curve due to its much wider use in first- and second-line treatments in later years. Lack of an alternative hypothesis drives the assumption that other molecules will retain their share in third-line treatment.

## Results

The results are reported according to each ARV used in each line of treatment, and we have reported data in terms of market share of each ARV in each year as well as the total number of patients using each of these ARVs. Raw data are available in the Supporting Information File (S1 FC).

Table 2 shows the total number of patients projected to use ART in each year and in each line of treatment from 2015 to 2025.

**Table 2:** Number of adults on treatment (million).

Treatment regimen	Year										
	2015	2016	2017	2018	2019	2020	2021	2022	2023	2024	2025
First-line treatment	14.0	15.7	17.4	18.9	20.2	21.3	23.8	24.0	24.1	24.2	24.4
Second-line treatment	0.6	1.0	1.4	1.8	2.4	2.9	3.3	3.3	3.4	3.5	3.5
Third-line treatment	0.0	0.1	0.1	0.2	0.3	0.4	0.5	0.5	0.6	0.6	0.6
**Total adults on treatment**	**14.6**	**16.7**	**18.9**	**20.9**	**22.9**	**24.7**	**27.6**	**27.8**	**28.1**	**28.3**	**28.5**

Data source used: UNAIDS Fast Track Report 2015 [4] and [10].

Of the total patients, each individual would be on one treatment regimen. Based on our assumptions, we have projected the market share of each ARV (Table 3). These data highlight the evolution of both the backbone and companion drugs for existing and future ART regimens.

**Table 3:** Forecast data showing proportion of adults using different ARVs as part of each line of therapy.

Regimens/Drugs Used	Year										
	2015	2016	2017	2018	2019	2020	2021	2022	2023	2024	2025
**1) First-line regimen**											
(i) Backbone drug 1											
AZT	22%	16%	9%	7%	4%	3%	3%	2%	1%	1%	1%
TDF	76%	81%	88%	90%	93%	92%	90%	85%	81%	71%	65%
TAF	0%	0%	0%	0%	0%	2%	4%	10%	15%	25%	31%
Other	3%	3%	3%	3%	3%	3%	3%	3%	3%	3%	3%
(ii) Backbone drug 2											
3TC or FTC	100%	100%	100%	100%	100%	100%	100%	100%	100%	100%	100%
(iii) Companion drugs											
NVP	26%	18%	10%	6%	2%	0%	0%	0%	0%	0%	0%
EFV	73%	81%	87%	89%	82%	78%	73%	62%	56%	50%	42%
DTG	0%	0%	2%	4%	15%	21%	26%	38%	44%	50%	57%
Other	1%	1%	1%	1%	1%	1%	1%	1%	1%	1%	1%
**2) Second-line regimen**											
(i) Backbone drug 1											
AZT	31%	40%	43%	45%	51%	59%	73%	68%	64%	61%	57%
TDF	61%	55%	49%	44%	35%	21%	1%	1%	0%	0%	0%
TAF	0%	0%	0%	0%	0%	2%	4%	6%	8%	8%	8%
DTG	0%	0%	3%	6%	9%	13%	16%	20%	23%	26%	30%
Other	8%	5%	5%	5%	5%	5%	5%	5%	5%	5%	5%
(ii) Backbone drug 2											
3TC or FTC	100%	100%	97%	94%	91%	87%	84%	80%	77%	74%	70%
(iii) Companion drug											
LPV/r	72%	63%	54%	50%	47%	46%	44%	41%	37%	32%	26%
ATV/r	26%	35%	44%	49%	51%	51%	52%	54%	57%	61%	66%
DRV/r	0%	0%	0%	0%	0%	1%	2%	3%	4%	5%	6%
Others	2%	2%	2%	2%	2%	2%	2%	2%	2%	2%	2%
**3) Third-line regimen**											
DRV/r	56%	56%	56%	56%	56%	56%	56%	56%	56%	56%	56%
RAL	70%	70%	65%	60%	55%	49%	45%	41%	37%	32%	28%
DTG	0%	0%	5%	10%	15%	21%	25%	29%	33%	38%	42%
Others	74%	74%	74%	74%	74%	74%	74%	74%	74%	74%	74%

### Market share of different ARVs in first-line treatment

Currently TDF (mainly with 3TC, but with FTC in 25% of the cases) is the most frequently used drug in the nucleoside/nucleotide backbone. Based on past data of WHO Survey on ARV Use, from 2009 to 2014 [11–16], its market share increased consistently at the expense of the market share of AZT, to reach 69% of people on first-line treatment by 2014, and is forecasted to continue increasing.

**Fig 2:**
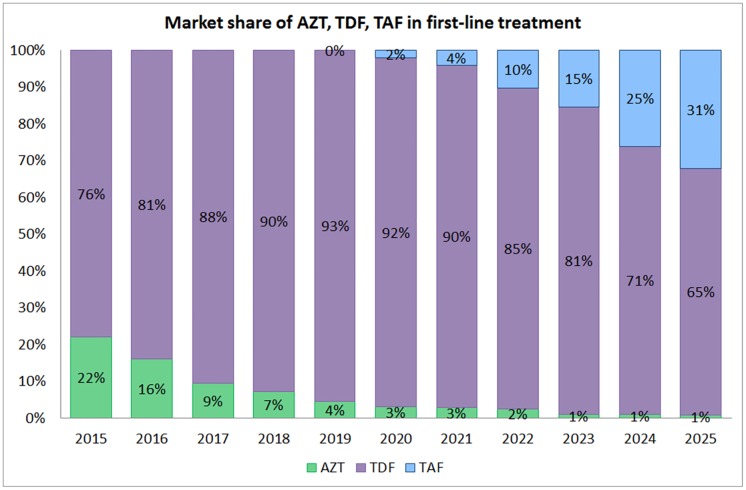
Market share of AZT, TDF and TAF in first-line treatment from 2015 to 2025.

As shown in Fig 2, our projections found that AZT market share will decrease to 4% by 2019 and then decrease further to stabilise at about 1% from 2023 onwards.

The introduction of TAF in 2019 will progressively erode the market share of TDF, leading to 31% patients on first-line using TAF by 2025. Other nucleoside analogues (such as d4T, ddI, and ABC), which, according to the WHO Survey on ARV Use 2015 [16] were used by 3% of adult patients in LMICs in 2014, and will continue to be used by increasingly fewer patients until 2025.

Whether TDF, AZT, or TAF is used, they will always be used with either 3TC or FTC, most often as part of a FDC product. TAF in combination of FTC will likely become available before its combination with 3TC, because Gilead Sciences (originator for both TAF and FTC) has obtained regulatory approval for the FDC of TAF/FTC. Owing to lack of clinical data on regimens containing TAF and 3TC and consequent uncertainty on development of FDCs containing both these drugs, we did not forecast the relative market share (proportion of FTC versus 3TC).

In first-line treatment in LMICs, using data from the WHO Survey on ARV Use 2015 [16], nucleosides/nucleotides were used in 2014 with either EFV (by 65% of patients) or NVP (by 35% of patients). All other possible companion drugs (including protease inhibitors, abacavir, integrase inhibitors, and other non-nucleoside analogues) accounted for only 1% of the total usage of ARVs in first-line treatment in LMICs in 2014.

**Fig 3:**
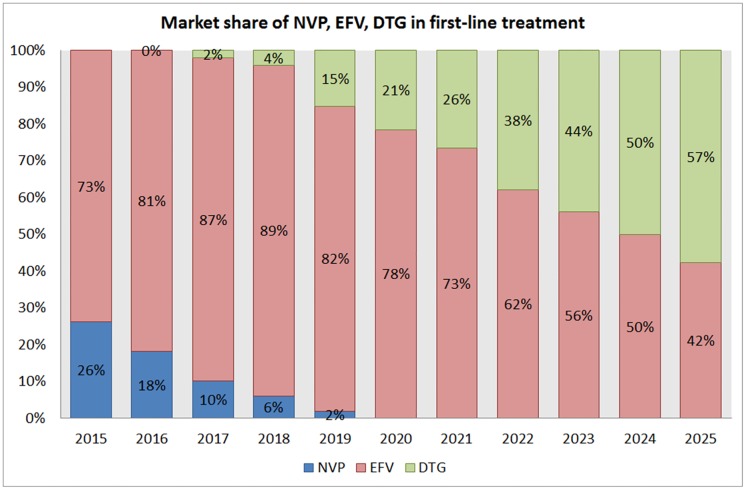
Market share of NVP, EFV, DTG in first-line treatment from 2015 to 2025.

In our model (Fig 3), following its generic introduction in 2016, DTG is projected to be used by 57% of PLHIV on first-line treatment by 2025. Following the WHO Guidelines on ARV Use 2016 [20], we predict that the use of NVP will decline to negligible by 2020. Other companion drugs will be used by 1% of the patients, but will not gain acceptance as their price will remain too high and/or their use relatively less convenient than DTG and EFV.

### Market share of different ARVs in second-line treatment

The uptake of second-line treatment in LMIC is limited: as per the WHO Survey on ARV Use 2015 [16], an estimated 5.3% of people taking ARVs were using a second-line treatment regimen in 2014.

**Fig 4:**
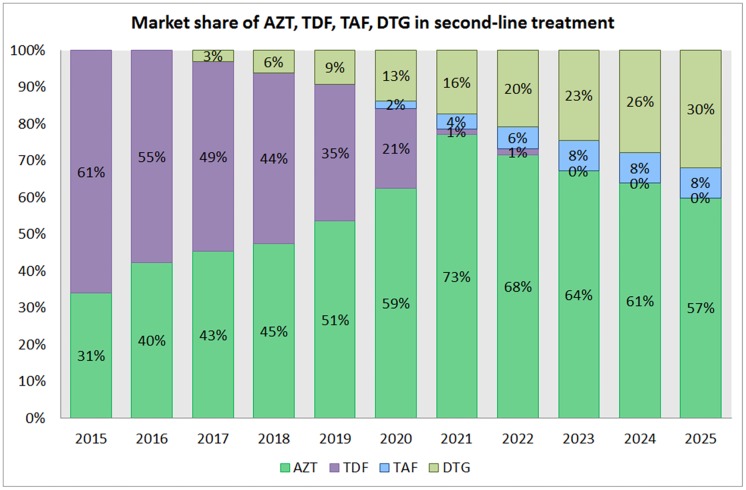
Market share of AZT, TDF, TAF and DTG in second-line treatment from 2015 to 2025.

Despite the fact that the WHO Guidelines on ARV Use 2016 [20] recommended the use of AZT in second- line treatment, in 2014 65% of second-line patients were using TDF in the backbone of their ARV regimen and only 25% were using AZT. Based on our model (Fig 4), we project that use of AZT will increase and be used in second-line regiments by 51% patients by 2025.

This is due to the higher use of TDF in first-line regimens and use of AZT after failure of first-line treatment. This explains the projected decline in use of TDF, which we calculate reaches 1% in 2021. Another reason for decline in use of TDF will be the availability of clinical data on use of DTG with protease inhibitors. We forecast that DTG, in combination with a protease inhibitor, will reach 30% of patients on second-line treatment by 2025, eroding the market share of nucleoside analogues. The slow uptake will likely be due to DTG’s higher use in first-line treatment and the fact that it is currently not recommended in second-line treatment in the WHO Guidelines on ARV Use 2016 [20].

**Fig 5:**
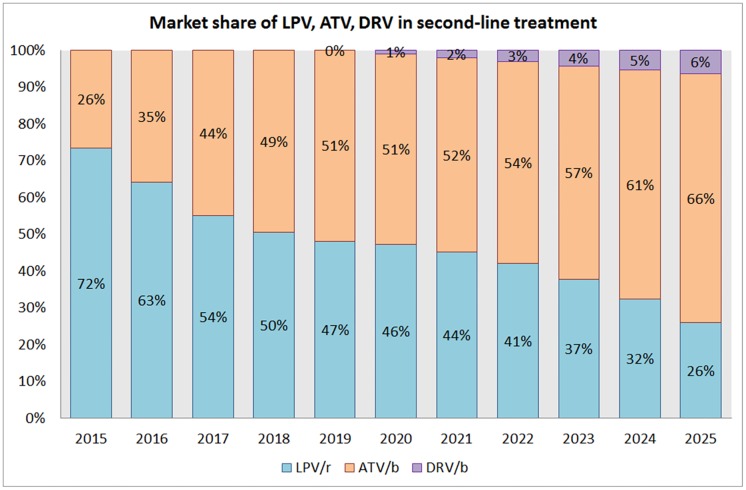
Market share of protease LPV, ATV, DRV in second-line treatment from 2015 to 2025.

As per the WHO Survey on ARV Use 2015 [16], LPV/r was the most often used companion drug in second-line treatment regimens, with 81% of patients in second-line treatment using it, and ATV/r was used by 17% in 2014. An increased uptake of ATV/r has been reported in the last two WHO Survey on ARV Use 2014 [15] and 2015 [16]. We forecast (Fig 5) an increase in use of ATV/r based on this data, reaching 66% market share by 2025.

We forecast that LPV/r will decline to 26% market share by 2025. We predict that DRV/r will play a limited role in second-line treatments, reaching 6% market share by 2025, because its price and lack of availability as low-dose FDC with ritonavir is a handicap compared to other protease inhibitors. It is worth noting that generic DRV/r FDCs are under development but pill size is expected to be very large. Eventually two half-dose pills may be developed, which will increase the pill burden over the current once daily dose of ATV/r.

### Market share of different ARVs in third-line treatment

**Fig 6:**
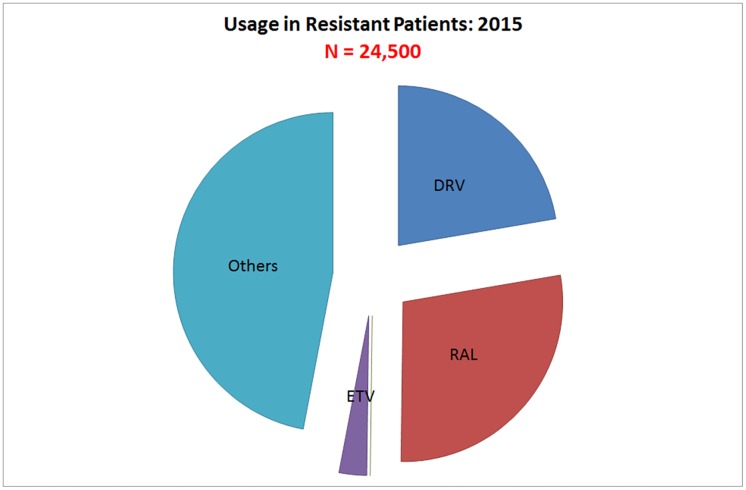
Market share of ARVs in third-line treatment: 2015 and 2025.

Third-line treatment is an area of considerable uncertainty, because at present it is estimated that its uptake in LMIC is limited to 26,000 adult patients. About a quarter of those patients use DRV/r, one third use RAL, and the remainder a variety of other ARV molecules. For our model (Fig 6), we have made the assumption that in relative terms this will not change much, except that DTG will take over market share from RAL. However, in light of the new developments towards increased uptake of viral load assessment in treatment monitoring globally [37,38], we have made the assumption that the number of people on third-line treatment will increase to approximately half a million by 2025.

### Number of people forecasted to take different ARVs

Data from the UNAIDS Fast Track report [3,4] predicts a demand of 24.7 million person-years of ART in 2020 and 28.5 million person-years of ART in 2025. Table 4 shows the total volume, in million person-years, of different ARVs forecasted to be used between 2015 to 2025: for first-line, second-line, and third-line treatments combined.

**Table 4:** Forecasted data showing number of people on each drug (million).

Drug	Year										
	2015	2016	2017	2018	2019	2020	2021	2022	2023	2024	2025
TAF	0.0	0.0	0.0	0.0	0.0	0.5	1.1	2.6	3.8	6.4	7.9
TDF	10.9	13.3	15.6	17.0	19.6	20.2	21.5	20.3	19.5	17.2	15.8
AZT	3.2	2.8	2.2	2.2	2.1	2.4	3.1	2.8	2.4	2.3	2.2
NVP	3.6	2.8	1.7	1.1	0.4	0.0	0.0	0.0	0.0	0.0	0.0
EFV	10.0	12.5	14.8	16.4	16.2	16.3	17.0	14.4	13.1	11.8	10.0
LPV/r	0.4	0.6	0.7	0.9	1.1	1.3	1.4	1.3	1.2	1.1	0.9
ATV/r	0.1	0.3	0.6	0.9	1.2	1.4	1.6	1.7	1.9	2.1	2.3
DRV/r	0.0	0.0	0.1	0.1	0.2	0.2	0.3	0.4	0.4	0.5	0.5
DTG	0.0	0.0	0.0	0.1	3.2	4.9	6.7	9.6	11.2	12.8	14.8
RAL	0.0	0.0	0.1	0.1	0.1	0.2	0.2	0.2	0.1	0.1	0.1

It is important to note that even with the early introduction of new drugs and assumption of their timely regulatory approval, the demand for existing drugs will continue to grow. From 10.9 million patients in 2015, the demand for TDF is expected to double to 21.5 million in 2021, where introduction of TAF could start eroding its market. Similarly, use of EFV will increase from 10 million patients in 2015 to 16.4 million in 2018, after which it will decrease with the arrival of DTG. Even with the arrival of the new drugs, by 2025, 15.8 million patients would be using TDF and 10 million EFV. These patient numbers will continue to increase because there will be a higher number of total patients on treatment [4].

In the case of new drugs (DTG and TAF), by 2025–9 years and 6 years post introduction of DTG and TAF respectively—these drugs will be used by 14.8 million and 7.9 million patients respectively as per our model. We report that the demand for DRV and RAL, although low, will still reach 0.5 million and 0.1 million patients respectively by 2025.

Based on our model, we report that in spite of a forecasted decrease of the market share of LPV in second-line treatment, its demand will more than triple from 0.4 million patients in 2015 to 1.3 million patients in 2020, and that of ATV will increase more than tenfold from 0.1 million patients in 2015, to 1.4 million in 2019, and 2.3 million in 2025. Demand for AZT and NVP will decline, reaching 2.2 million and 0 million patients, respectively, by 2025.

## Discussion

The UNAIDS Fast Track report [4] has predicted 24.7 million PLHIV on ART in 2020 and 28.5 million PLHIV on ART in 2025. Of this, 24.3 million would be on first-line, 3.5 million on second-line, and 0.6 million on third-line treatment by 2025. Our data has highlighted that that DTG and TAF are expected to be major players in the ART in the next decade, with 15 million and 8 million PLHIV using these ARVs respectively by 2025. However, as ART scale-up is predicted to increase considerably in the next few years and there are some concerns on safety and efficacy of DTG and TAF during pregnancy and among TB/HIV co-infected patients using rifampicin, there remains a clear need for continuous supplies of existing ARVs including TDF and EFV in future as well, which we predict 16 million and 10 million patients—respectively—will be using in 2025. It will be important to ensure that the existing capacities of generics manufacturers, which are geared towards ARVs of higher doses such as TDF 300mg and EFV 600mg, will not be adversely impacted due to the introduction of lower dose ARVs such as DTG 50mg and TAF 25mg. At the same time, more patients would be on second-line treatment due to the higher use of viral load testing and LPV/r and ATV/r will be used by 1 million and 2.3 million patients respectively by 2025.

The most critical assessment for supply security is what the assumptions used for first-line treatment are, because approximately 95% of all people in LMIC are currently using first-line treatment regimens [2]. We made the assumption that new ARVs such as DTG and TAF could be introduced in first-line treatment as soon as (a) they are recommended in WHO Guidelines on ARV Use and (b) affordable generics versions of those ARVs become available. Because the Medicines Patent Pool is the main broker of voluntary licenses for those drugs to generic producers and has access to information on the planned product launches, we are confident that the information on the timing of their availability is correct. In addition, the Medicines Patent Pool has insight in the future targets for their price through regular meetings with key manufacturers, and is able to independently confirm that the price advantage that they would have over presently used drugs is valid.

We assume the newer ARVs will also likely significantly reduce the cost of treatment over time, considering the lower dosage of these ARVs. Given the decreasing funding available for ART, this would likely help in ensuring more people are put on treatment to meet the UNAIDS projections—leading to a higher trend of use of new ARVs such as DTG post introduction as compared to TDF in the past. Hence, it will remain important to continue monitoring and forecast the development of the ARV market in LMIC to guarantee the availability of the ARV medicines needed.

### Limitations

We acknowledge that although our forecasts include a mix of data and several of our assumptions are judgement calls and can be challenged. Whether the UNAIDS Fast Track scenario [4] for total number of patients on ART, which we used as our estimate of ART demand, will become reality—is unknown. However, our predicted demand of 24.7 million person-years of ART in 2020 and 28.5 million in 2025 is in keeping with the publicly available estimate of future ARV demand produced by UNAIDS [4]. In addition, there are limitations in our approach in terms of our calculations for second-line treatment, and consequently salvage treatment. We base our forecast on UNAIDS data [3,4] suggesting that by 2018 approximately 10% of people would be on second-line or third-line treatment, because more people will be able to access it when viral load is increasingly used to monitor treatment efficacy. This assumption may be optimistic in the short-term, because at present the average number of viral load tests done in LMICs is 0.5 tests per person per year [37–39]. However, countries that have already scaled-up viral load testing have increased the proportion of people on second-line treatment, and countries like Botswana, Brazil, and South Africa are increasingly making third-line treatment options available to their population. It is important also to consider is the time of introduction for new ARVs and their respective market share over the next 10 years, and we believe that our assumption of replacement of older molecules with new ones is a valid approach.

### Next steps in ARV uptake: ensuring alignment of all stakeholders

Our data highlight that the availability of new ARVs DTG and TAF are expected to lead to major changes in the proportion of ART regimens used in LMICs as early as 2025. The timely availability and affordability of these new ARVs will be crucial in order to avoid treatment interruptions and procurement agents should be gearing up for the increased number of PLHIV on treatment as well as the new ARV medicines in the market.

As highlighted in Table 5, multiple pharmacokinetic and clinical studies are underway or being planned for DTG and TAF. Completion of these studies will further inform and strengthen our assumptions around ARV availability and volumes, especially the pharmacokinetic studies for use of TAF in TB/HIV co-infected patients. Any delay in the pharmacokinetic study would adversely affect the introduction of TAF in LMICs and also its uptake in the coming years.

**Table 5:** Summary of the key clinical studies on DTG and TAF in adults.

Study	Sponsor	Study arms & patient population	Main objectives
NAMSAL Study ANRS 12313; Phase III; Cameroon	Inserm-ANRS	TDF/3TC/DTG vs TDF/3TC/EFV400 for treatment-naïve adults	Non-inferiority of DTG vs EFV400 when combined with TDF/3TC as first-line treatment for adults in resource-limited settings (RLS)
ADVANCE Study; Phase III in planning; South Africa	Wits RHI	TAF/FTC/DTG vs TDF/FTC/DTG vs TDF/FTC/EFV600 for treatment-naïve adults and adolescents ≥12 years	Non-inferiority of TAF/FTC/DTG when compared with TDF/FTC/DTG or TDF/FTC/EFV600 as first-line treatment for adults & adolescents in RLS
IMPAACT Study 2010; Phase III in planning; Multiple sites including RLS	NIAID IMPAACT Network	TAF/FTC/DTG vs TDF/FTC/DTG vs TDF/FTC/EFV600 in treatment-naïve pregnant women (other than prior PMTCT) from 14-28w gestation, and their infants	Superiority of DTG-containing regimens vs TDF/FTC/EFV600 for virologic suppression at delivery in pregnant women and their infants. Safety of each of the 3 regimens (adverse pregnancy outcomes, and maternal and infant adverse events)
DolPHIN1 Study; Phase II/III; Uganda	University of Liverpool, in collaboration with ViiV and Makerere University	TDF/3TC/DTG vs TDF/3TC/EFV600 for untreated pregnant women (≥28 -36w gestation) and their infants	Safety and PK profile of DTG during the third trimester and post partum. Secondary outcome measures also include infant DTG level and proportion of mother-to-child transmission
TAF-RIF PK study; Phase I in planning	St Stephens AIDS Trust	TAF/FTC alone, followed by TAF/FTC with rifampin (RIF) co-administration, followed by TDF/FTC alone in healthy volunteers	The impact of RIF on the level of tenofovir diphosphate (the active metabolite) from TAF. Implications for use of TAF and RIF during treatment of HIV/TB co-infections
Study 117175; Phase III; Multiple sites including RLS	ViiV Heathcare	DTG50 twice daily + 2NRTIs vs EFV600 once daily + 2NRTIs during RIF-containing TB treatment in treatment-naïve adults	Efficacy and safety of DTG- and EFV-containing regimens in HIV/TB co-infected patients
ING200336 study; Phase III	ViiV Healthcare	HIV+ women on the ABC/3TC/DTG arm who become pregnant during Study NCT01910402	Safety and PK profile of DTG during pregnancy and post partum

Data Source: U.S. National Institutes of Health [40] and HIV/i-Base and TAG, 2016 Pipeline Report [41].

It is also clear that use of existing drugs such as TDF and EFV will remain high and manufacturers must not decrease manufacturing capacities of these existing drugs in the near future as the new range of low-dose ARVs come to the market.

## Supporting Information

S1 FC (XLSX)
